# Overcoming Childlessness: Narratives of Conception in Early Modern North India

**DOI:** 10.1017/mdh.2024.43

**Published:** 2024-10

**Authors:** Sonia Wigh

**Affiliations:** University of Cambridge, Cambridge, UK

**Keywords:** Reproductive medicine, Obstetrics and gynaecology, History of medicine, Mughal India, Generation

## Abstract

This article discusses early modern North Indian ways of expressing how barrenness could be mapped onto a woman’s maternal identity. Scholars have engaged with the historical evolution of women’s identities, focusing overwhelmingly on their economic and political potential. This article is the first to use medical and erotological sources from the seventeenth and eighteenth centuries to study women as procreative agents, and the socio-sexual anxieties prompted by infertile female bodies. Through a critical study of a wide range of medical material, I demonstrate that by the eighteenth century, several transformations in medical discourses can be mapped onto textual transmissions from Sanskrit (and Braj Bhasha) to Persian, as well as between competing but conterminously flourishing medical paradigms, Ayurveda and Yunani. While cures for childlessness have a much longer history, a new genre of ‘anonymous’ sources, particularly focused on the sexual diseases of men and women emerged in early modern North India. Lastly, my comparative methodological approach to different textual genres will complicate our understanding of early modern medical episteme and its intended audience.

The kingdom of the heavens and earth belongs to God. He creates what He pleases, for some He grants females, for some He grants males, for some He grants males and females, and some He makes childless. He is wise and capable.

- (The Qur’ān, 49:50)^1^

Until my father [emperor Akbar] was twenty-eight years old, none of his children had survived and he was always soliciting dervishes and hermits (who have spiritual proximity to the divine court) for the survival of a child … During the days when my exalted father was seeking a son, (he met) an ecstatic dervish named Shaykh Salim … Because my father was a devotee of dervishes, he visited this one too. One day while Shaykh Salim was in a trance, my father asked him, ‘How many sons will I have?’

‘He who bestows without obligation will grant you three sons,’ he replied.^2^

Childlessness and the consequent desire for progeny were concerns deeply rooted in early modern North Indian society. This paper will focus on how diverse issues surrounding women’s fertility were discussed in early modern North India across three broad genres: *ṭibbī* (medical, ranging from theoretical medical manuals to clinical notes of practising physicians); erotological; and early modern literary production in Persian, Braj Bhasha, and Urdu.^3^ Through a critical analysis of these sources, this paper demonstrates that even though yearning for a child continued to be an early modern societal concern, it wedded the experience of child-bearing to the idea of femininity itself. To gain such an experience, women used the help of male and female medical professionals and sought divine interventions.

Scholars like Lal and Balbanlilar have demonstrated the primacy of reproduction in an elite woman’s life, especially as it became ‘vital for the survival of the empire that was coming into being’ by the early seventeenth century.^4^ The Timurid-Mughal empire spanned three centuries (1526–1857) and covered vast geographies controlled through a layered imperial revenue system.^5^ This stimulated commercialisation of the economy and, coupled with increased participation in the seventeenth-century global trade, led to the concentration of wealth and power in the subcontinental nobility’s hands. Apart from immigrants from Central Asia, Iran, or (present-day) Afghanistan, the Mughal emperors incorporated many local rulers into their military-administrative order and imperial services. These imperial functionaries became major landholders (*mansabdars*), revenue collectors, commandeered soldiers, and important patrons of arts and architecture.^6^ These imperial land grants were not routinely hereditary and birth defined one’s initial ranking, entry into courtly circles, and subsequent career. While patrimonial alliances defined and shaped an individual’s identity, in the early days of the empire, ‘maternal origins were reflected in the status of princes, and Timurid children were often given names from their matrilineal line, which had a permanent claim on their loyalty and critical role in shaping their identity, as well as that of their mothers’.^7^ Over the next hundred years, the Mughal harem saw substantial changes, especially through the influx of women with Shi‘i, Rajput, and even Christian ancestries within their ranks; however, the desire to produce a child, an heir, persisted.^8^ Concomitantly, the inability to produce a child became a source of grave anxiety not only for the affected woman but for society as a whole.

Most elite households, in accordance with practices in the Mughal harem, fostered hierarchies amongst women. These were primarily based on their origin, marital status, concubinage, slavery, and the ability to bear children. The Timurid-Mughal practice of tracing the legitimacy of rule through descent from both the mother’s and father’s families has been well established.^9^ If a woman was unable to bear children, the practice of fostering, especially through inter-familial adoption, is also well-documented.^10^ Furthermore, the birth of a child was of primary importance to a woman, especially when seen in conjunction with the social equity it provided her. According to the Ḥanafīs, the most popular of the four religious Sunnī Islamic schools of jurisprudence (*fiqh*) in early modern South Asia, even a slave wife’s status would change in the event of carrying her master’s child, thereby making her *umm walad* (mother of the child). She could not be resold, whereas theoretically, other slave wives could be.^11^ And yet, producing a child was not the summation of a woman’s existence, as evinced by numerous examples of women being politically active, economically independent, and producing and patronizing courtly literature.^12^ While I acknowledge that a woman’s role in empire-building was beyond reproduction, and contingent on more than producing or fostering a male heir^13^, this article will argue that early modern erotological and medical texts were geared towards fostering reproduction, in as much as they viewed the inability to bear a child as one of the main ‘special diseases of women’.^14^ Clearly, a child, an heir, was instrumental not only to secure a man’s fortune but to also secure the position of the woman as an important member of the household, not only in her husband’s lifetime but also in her son’s. This article is an exploration of fertility within this matrix of wealth and inheritance.

## Sources and methodology

The Mughal Empire was one of three principal Islamic empires of the early modern period, the others being the Safavids in Iran and the Ottomans in West Asia and Eastern Europe.^15^ The Mughals had their ancestral domains in Central Asia, where political and familial turmoil led them to seek other avenues. After their settlement in India and absorption of the pre-existing court cultures, Mughals patronised and facilitated the development of an enormously rich South Asian variation of ‘Persianate’ culture by encouraging, among other things, the in-migration of poets, artists, physicians and other professionals from an increasingly sectarian Safavid Iran.^16^ The culture of the Mughal court has often been labelled ‘Indo-Persian’, signifying firstly a synthesis of ethnic, cultural, and political elements from India, Iran, and Central Asia, and secondly the use of Persian as a pan-Indian, courtly, and quotidian language. My paper is set in this Indo-Persian milieu. This is not a story of seamless amalgamation but of the transformation of languages, (medical) ideas, and identities. The sources discussed include texts created within the realm of Ayurvedic medical traditions in Sanskrit and the transformation of these ideas when translated into Persian, perhaps for an audience better acquainted with the Greco-Arabic Yunani paradigm. Scholars like Askari, Speziale, and Alavi have shown that the composition of many Yunani works can be owed to the impetus provided by the imperial Mughal state’s interest in understanding the pre-existing medical cultures of the sub-continent and utilizing them to create a thriving medical tradition.^17^

Scholars such as Shah and Selby have demonstrated that ancient and medieval medical discourses in South Asia, primarily in Sanskrit, were deeply interested in issues of conception, gestation, and childbirth.^18^ By the early modern period, this focus on restoring and promoting fertility remained unchanged, hence I will explore subtle transformations in *how* the discussion of disease and curatives was translated and brought into the Indo-Persian knowledge paradigm. Furthermore, the investment in treating female infertility is not new, as evident from the plethora of sources including medical, erotological, literary, and biographical, that have included snippets of medical-dietetic information on the subject. The difference also stems from a whole range of ‘new’ texts that emerged in the early modern period: succinct, mostly untitled, and anonymously authored texts about sexual diseases of men and women that were produced between the mid-and late eighteenth century. These texts move beyond the strict, theoretical boundaries of *ṭibbiyā* texts to provide simpler prescriptions, indicative of a broadening medical understanding as well as the possibility of self-diagnosis and treatment. They also make more space for the inexplicable, divine, and occult to remove impediments to infertility. Through a critical analysis of how barrenness was treated in these texts, I also speculate on the nature of their anticipated audience, the extent of the audience’s medical knowledge, and inter-textuality with other more established medical genres.

By exploring the myriad ways in which these texts deal with issues associated with fertility, I will also showcase different medical perspectives on reproduction and regulation of the female body. Here, fertility, measured as a woman’s ability to produce offspring, forms a major concern in early modern medical and literary traditions. Using a comparativist approach, this paper will choose one kind of problem that leads to female infertility and follow its treatment across two major textual genres: erotological and medical. I will also demonstrate that the medical or *ṭibbī* genre is not monolithic and has three sub-genres: *Qarābadīn* [pharmacopœia(s)], and theoretical manuals that deal with each disease from its treatment from head to toe; *Maṭab*(s) or case files of physicians (*ḥakīm*); and lastly the most ephemeral category from the end of eighteenth century: smaller, incomplete, mostly anonymously authored texts, focusing on sexual diseases.^19^ A cursory glance at some of the indicative titles such as ***ʿ**Ilaj-i **ʿ**aurat* [Cures of women] and *Risāla dar **ʿ**aurat* [Treatise about women] reiterates that they were focused on the diseases, especially reproductive ones, of women. By comparing these varied texts, I demonstrate that the same set of problems can be dealt with very differently in contemporary sources even though there is a great deal of intertextuality. Furthermore, the ubiquity of sources offering prescriptions to cure infertility is indicative of the deep-seated anxieties around childlessness in early modern North India.

## Difficulty in conceiving: Medical help to produce a child

The biographical account of a high-ranking Mughal judicial official named Khwaja Dost Muḥammad, who was active in the reign of the Mughal emperor Jahangir (r. 1605–27), contains an interesting story of a case he adjudicated, where a man accused his deceased brother’s wife of fraudulently passing off a child as his brother’s offspring to lay claim to his property. The dispute was founded on his declaration that his now-departed brother was impotent and could not produce a child with his wife in his lifetime. Khwaja Dost Muḥammad is said to have summoned the mother and the child and made inquiries. The woman acknowledged that there was something deficient in the virility of her husband (*dar rujūlīyat shauhar-i man nukṣānī būd*), but a physician (*ḥakīm*) had advised her to give her husband *rohu* fish including its head to eat for a period of forty days without any break, which would make him virile. By diligently following the *ḥakīm*’s instructions, the man’s virility was restored, and a son was born to them. Furthermore, the Khwaja tested the paternity of this child by the smell of fish in the sweat that emanated from the child.^20^ The report of this incident brings forth a possible scenario of how the courts dealt with ascertaining a charge of male impotence, even though made posthumously, and intimately connected it with issues of rightful inheritance.^21^ Here the onus of proving that her child was a legitimate progeny fell on the woman.

Furthermore, the solution applied by the Khwaja gives us insight into how certain food items, not necessarily complex medicaments, were indelibly linked to curing infertility. The ‘check’ by the *Khwaja* to ascertain the child’s parentage demonstrates judicial authorities utilising popular but informed ideas to make legal decisions. As for the nature of the check itself, it involved ascertaining whether the qualities of the medicine or method used to restore virility to the father making the child’s conception possible were retained and manifested in the resultant progeny.^22^ The incident also depicts the resourcefulness of the woman in curing *her* childlessness by seeking a cure for *his* infertility. She clearly stated that even though her husband had been impotent for a long time, she sought help from a *ḥakīm* who ‘cured’ him by ordering a diet of *rohu* fish and its head for forty consecutive days. The physician’s solution, a specific course of regimentation of diet to restore virility, was clearly situated in the contemporary Yunani humoural understanding that *rohu* fish was an aphrodisiac, albeit a rare and expensive one. Even in contemporaneous Ayurvedic traditions, it was supposed to be ‘hot, heavy and, moist … it is aphrodisiac. It promotes strength’.^23^ Building a link between the fish’s appearance and its medicinal properties, in the *Suśruta-saṃhitā* the shape of the uterus is likened to a *rohita* (*rohu*) fish, which would explain why it was used as a curative for feminine reproductive issues.^24^

The fish was much sought after in the imperial household too. For instance, in his memoirs, Jahangir mentioned his affinity for fish several times, especially *rohu.* ^25^ The use of fish as a treatment for infertility seems to cut across religious beliefs and food practices. A similar incident was reported by the French Protestant traveller, Jean-Baptiste Tavernier (1605–89). He noted that the wife of a rich *bania* merchant, Shantīdās, in Ahmadabad (Gujarat), could not conceive for more than fifteen years and was discreetly advised by the domestic help working in her house, to eat three or four ‘little fish’. For most upper-caste Hindus, who usually practised vegetarianism, religious prohibitions created issues, so the servant disguised it in a manner that she could consume it, followed by sexual relations with her husband.^26^ Her husband passed away as she became pregnant. Following a property dispute, she approached the Governor, but the matter escalated to the Mughal emperor. Both ordered the newborn to be given a bath, as the bathwater smelled of fish it was proven that the remedy for curing infertility used by the woman was genuine.^27^ Both stories not only indicate that fish was commonly held as a ‘popular’ cure for infertility, both for men and women but also show that issues of infertility were significant in solving property disputes and fed into the making and articulation of early modern sexual and social anxieties.

This almost exclusive focus on female reproductive abilities draws on the theoretical, moral, and religious underpinnings of women’s medicine in early modern South Asia. Through this paper, I demonstrate that even though most medical (*ṭibbī*) and erotological texts discuss the same sets of diseases and curative practices, texts produced in different sub-genres bring their own complexities to the issue, which reveal as much about the spatial and material setting of cures of feminine diseases, as about the audience of these texts.^28^ Methodologically, a critical analysis of the medical treatments offered to infertile women in different types of texts leads to explorations of who was offering explanations and treatments for ill health, as well as their relationship with the intended patients. Much like us, early modern patients could (and did) seek remedies across medical systems with differing humoral understandings of health, such as Ayurveda and Yunani. While Ayurveda is premised on the doctrine of the three humours that regulate the state of the body – wind (*vāta*), choler (*pitta*), and phlegm (*kapha* or *sleṣman*) – the four-fold humoural system followed by Yunani texts embodying the spirit of individual well-being included blood (*dam*), phlegm (*balgham/baljam*), yellow bile/choler (*ṣafrā’*), and black bile/melancholy (*saudā’*).^29^ Many medical theoreticians attempted to study, translate and collate information from these traditions (and even reconcile these humoral differences) and present them through detailed translations.^30^ Through a critical study of material that has been translated within these two humoral models, as well as their associated Sanskrit and Persian linguistic traditions, this paper will not only demonstrate complex networks of early modern intertextuality but comment upon the primacy of reproduction in discussions centered around diseases of women.

## Eroto-medical discussions of barrenness in the *Kokaśāstra* and *Lazzat al-nisā’*

The first section examines how barrenness was understood and depicted in detailed medical sections of erotological texts. The primary source for this section is a specific cluster of eroto-medical texts titled *Kokaśāstra* [Teachings of Koka Pandit], and *Laẕẕat al-nisā’* [The Pleasures of Women]. I am consciously using the term ‘eroto-medical’ as these texts not only deal in detail with sex and pleasure but also with sex and reproductive health. These texts covered many subjects including (but not limited to) physiognomy, astronomy, and literary and ethical discussions. Sometimes they demonstrate an excessively canonizing intent by creating the idealized categorizations of men and women and their descriptions based on sexual characteristics which were shared across genres and even languages. There is a limited amount of scholarship on *Laẕẕat* and what exists is generally based on catalogue entries made by linguists, who classify this as a mere Persian translation of the *Kokaśāstra.* ^31^ The Sanskrit *Ratirahasya*, also known as *Kokaśāstra*, was composed by the author Kokkoka, also named Koka or Koka Paṇḍita, in the thirteenth century.^32^ As is expected, while *Laẕẕat* acknowledges that it was meant to translate (*tarjuma kardan*) or compile (*taṣnīf*) the knowledge of Koka Paṇḍit into Persian, the author added several pieces of advice (*ḥikmat*) borne from his knowledge or experience. By the mid-seventeenth century, *Laẕẕat al-nisā’* emerged as a multi-genre, multi-form, and multi-functional text in Persian. One of the earliest extant copies of the text, attributed to Muḥammad Shah ‘Jāmī’, is dated c. 1646 and was written entirely in verse.^33^ He dedicated it to his patron, the Sultan of Golconda, **ʿ**Abd Allāh Quṭb Shah (r. 1626–72).^34^ This text was not only a handbook for (theoretically) finding ideal partners, enhancing sexual pleasures, and providing medical cures, but rather fulfilled the larger purpose of instructing women to act as intimate companions for elite noblemen. It was copied several times, and its form changed significantly by the early eighteenth century by which time it was usually in prose and the medical sections dominated the content.^35^

Within this context of multilingual translations, I will start by looking at a short section in the Sanskrit *Kokaśāstra* titled ‘Recipes for pregnancy’ that mentioned a few cures to counter barrenness, one of which was:

If a woman on the day of the end of menstruation drinks the powdered root of *Navanagakesara* [sic] with ghee and milk and then unites with her husband, she becomes pregnant.^36^

This seems like a simple ingredient-based remedy to aid the removal of any defects that eventually hindered the conception of a child. All three ingredients used in the recipe have properties ensuring that there were no blockages in the digestive system of the body. *Nāgakesara* (*Mesua ferrea*) was consumed as a stomachic to promote appetite or assist digestion and cure any irritability of the stomach.^37^ Ghee and milk were also considered laxatives, which facilitated the power of digestion, eventually removing all impediments to conception. *Nāgakesara* was a rare and expensive product, not available for common use.^38^ A much more common and easily available ingredient was *ghee* prepared from cow’s milk, meant to increase the power of digestion, increase seminal fluid, and remove impediments caused by phlegm (*kapha*) and fat (*medas*).^39^ A similar role was played by milk, which is meant to be life-giving and promote cooling, longevity, virility, and digestion. Clearly, all the ingredients were meant to be conducive to opening obstructions that prevented conception. Furthermore, as this was meant to be consumed after the woman’s menstrual cycle was over, it could possibly promote the production of seminal effusion when she was fertile.^40^

By contrast, when dealing with the issues of barrenness in *Laẕẕat*, Jāmī creates a complex narrative to explain the causes and ways to overcome impediments to establish the foetus in the womb. Signalling a departure from its *Kokaśāstric* antecedents, Jāmī created a back story about why and from where this knowledge is acquired. He wrote that once some women went to the Prophet Solomon (*Sulaiman Rasūl*) and asked him why they were barren or unable to have children (*farzand nayad az mā*)^41^. Solomon redirected the query to a *parī* or *jinn* (fairy or spirit) who listed seven reasons why a woman cannot conceive.^42^ It is noteworthy that Jāmī invokes Solomon, the wise ruler associated with *ḥikmat* or wisdom.^43^ It seems to be a literary device to derive legitimacy from a wise character, much like European texts sometimes spuriously referenced Aristotle. By the eighteenth century, it seems that erotic-medical texts like *Laẕẕat* increasingly focused on the medicinal sections, which became larger and more elaborate as the functionality of the text evolved. Jāmī explained each problem succinctly by creating a specific template. First, he mentioned the affliction, followed by the distinctive symptom (‘*alāmat*) of that problem in a woman’s body. Usually, the distinctive sign for each iteration of the disease was pain in a certain body part of the woman after sex. This was followed by the precise cure to combat the disease, including the ingredients and their quantities. Most of the cures were meant to be applied externally on the vulva with the help of cotton pads. After adding further instructions (as discussed below), he added an annotation to give thanks to God for his mercy. The following section from *Laẕẕat* discusses the sixth problem responsible for barrenness in women, the coldness of the womb:

**Table 1:** 

*Shashum, sard bāshad raḥim az zenān*,	Sixth (problem) is coldness in a woman’s womb,
*az sard-ish tukhm neh band dar ān.*	From the cold, there is no seed (*tukhm*) therein.
*bad-ān **ʿ**aūrat az b**ʿ**ād inzāl mard*,	With that, after the woman receives the male seminal fluid (*inzāl*),
*be-gūyad keh sīneh shawad pur az dard.*	(She) says her bosom is full of pain.
*shināsī keh sard-ish ghālib buwad*,	Knowing that the coldness is predominant,
*be-būsideh wa tukhm ẓay**ʿ**a shawad.*	(both) kiss, and seed is wasted.
*Bīkh wa panj wa barābar siyah dāneh khūb*,	Roots(?), and five (and) equal parts good fennel flowers,^44^
*siyah zireh har chār* *yak-jā be-kūb…*	(and) black cumin, for cure strike all four together…
*Be-jōshān tū dar tel kunjid chunān*,	You should boil them in sesame oil,
*Keh tā nīm ser az rōghan buwad.*	(take) half portion of that oil.^45^
*Pas az ḥaiẓ-i zan fikr-i shāfa buwad*,	After her menstruation, the woman must consider using this suppository,
*Dar ān tel wa panbeh tar wa shāfa kun.*	After that saturate the cotton pads with oil …
*Shawad basteh āb-i mardī raḥim*,	The seminal fluid of the man will be tied/locked inside the womb,
*shawad dūr az ranj-i sardī raḥim.*	the suffering caused by the cold womb will be removed.^46^

Here, Jāmī explains how coldness in the womb overpowers the seed (seminal fluid), which renders it wasted. The symptom of this type of disease is post-coital pain in the chest (*sīneh dard*). Jāmī urges the (presumably male) reader to neither give her a kiss, (alluding to sex) nor his seed as they will be wasted. By highlighting that ‘there is no seed therein’, Jāmī implies that the woman’s cold womb does not release the seed necessary for conception. Furthermore, if the womb is cold, she cannot feel or experience sexual pleasure, as heat is essential for desire and pleasure to be felt.^47^ This reaffirms that even though ‘traditionally’ not considered medical, early modern eroto-medical texts like *Laẕẕat* acknowledged that men and women both have seminal effusion, indicating that it operated firmly within the two-seed model of conception.^48^ The cold womb meant that the man’s seed could not mingle with the woman’s seed, as it was not released so no conception would be possible. The solution to combat the coldness was to create a suppository by pounding the black cumin (*siyāh zīrah*) and fennel flower (*siyāh dāne*), to be boiled in sesame oil. After this mixture was created a specific amount was to be soaked in cotton strips (*puṃbeh shāfeh*) and applied to the woman’s genitalia after menstruation. All these ingredients have heating properties and would remedy the womb’s coldness, the primary cause of the disease. The use of *zīrah* is to be expected as it was well-known for its ‘hot’ nature. It was also known for being a carminative (relieves flatulence), stomachic (promotes digestion), and astringent (shrinks certain body tissues).^49^ Fennel was also known as a hot and dry drug, with mild properties that helped digestion and strengthened, especially during coitus.^50^ Essentially, apart from their heating properties, all these ingredients aided digestion and together worked to restore health to the woman’s body. The effect was to ensure that the coldness be removed from the womb when the male seed (*āb-i mardī*) reaches it, leading to the successful formation of the foetus (*nuṭfeh*).

The literary structure created by Jāmī – symptoms, diagnosis, and cure – seems to be an abbreviated version of detailed theoretical *ṭibbī* texts (as discussed in the following section), which may be indicative of some measure of intertextuality amongst the *Laẕẕat* and *ṭibbī* textual traditions. This suggests that erotological verse narratives were closely influenced by the medical tradition and vice-versa. These texts included precise information that was pertinent to the reader. While it is difficult to ascertain if women were also reading erotological texts like *Laẕẕat*, the fact that an entire chapter was dedicated to treating feminine infertility reiterates that was a substantial enough feminine concern in the early modern period that medical solutions were compiled outside of strictly medical texts to combat it. Furthermore, the author did not include mere platitudes or invocations to God but gave specific diagnostic and curative solutions based on contemporaneous medical paradigms, indicating that the reader (or final consumer of this knowledge) either had the medical knowledge or at least informed awareness of recognising outward, obvious signs of disease and creating cures. While different texts propounded different prescriptions, the eventual aim was to facilitate the formation of the foetus and, subsequently, the successful completion of pregnancy. The next section explores how medical handbooks, primarily produced, and consumed by physicians engaged with issues of barrenness.

## Theoretical medical discourses: Curing the cold womb in the *Ṭibb-i Akbarī*

The history of the transmission of Greco-Islamic medical knowledge in Arabic and Persian can be traced back to the Delhi Sultanate (1206–1526), the patronage provided by the Mughal emperors amplified the study, practice, and diffusion of knowledge of *ṭibb* in early modern South Asia.^51^ One such text is the *Ṭibb-i Akbarī* [Medical Knowledge of Akbar (Arzanī)], a medical compendium which contained chapters on ‘special’ (*makhṣūṣ*) diseases of men and women. The *Ṭibb-i Akbarī* was composed by Ḥakīm Muḥammad Akbar Arzānī (d. 1722) in c. 1700–01. It draws heavily on the fifteenth-century Arabic text by al-Kirmānī titled *Sharḥ al-Asbāb wa al-‘Alāmat* [A Commentary on the Aetiology].^52^ Arzānī explicitly refers to Ayurvedic medical authorities such as Ćaraka and Suśruta, who had inspired Arabic medical writers from the seventh century.^53^ The text contains twenty-seven chapters, each chapter dedicated to diseases of body parts from head to toe. The language of *Ṭibb-i Akbarī* is prescriptive in function. Most verbs are conjugated in the optative mood, causative construction, or a sort of aorist tense in which a prescriptive function is understood.^54^ In most chapters, the text presumes the diseased body to be male, except in the chapters on the ‘diseases of the breast’, ‘special diseases of women’, and specifically marked sections and prescriptions for women. Within the chapter on special diseases of women, Arzānī looks at feminine health exclusively through the prism of how to prevent an abortion/miscarriage and any other impediment to a woman’s generative function. Interestingly, in the nineteenth chapter on the ‘special diseases of men’, the entire discourse ranging from the prognosis of the diseases, and symptoms, to the treatments, is defined by the intrinsic root of the problem: lack of desire or penile problems. Even linguistically, in *Ṭibb-i Akbarī*, when the discussion is centred around male impotence, the terminology used revolves around increasing sexual power and vigour (*quwwat-i bāh*). On the other hand, more often than not, when discussing infertility, the terminology used revolves around ‘*inzāl’* (seminal ejaculation). Seminal fluid was seen as the source of male virtue, the erection as a physical manifestation of an individual’s masculine libido and strength. A simple glance at the focus put on understanding the reasons behind impotency and finding appropriate cures for it in a range of medical texts shows how large a threat it was to early modern South Asian concepts of manliness.^55^ And yet, there were several other markers of masculinity and childlessness that did not become enmeshed with the masculine identity in the way it did for their female counterparts.^56^

At the beginning of the section describing the reasons for barrenness (***ʿ**aqr*), Arzānī acknowledged that it could be attributed to problems with men as well as women. Following the previously discussed example from the eroto-medical *Laẕẕat al-nisā’*, I will now discuss how ‘coldness of the womb’ as a cause for barrenness was understood in a handbook of medicine:

Type first: if the ill temperament that leads to the demise of the foetus is cold (in nature). And the seminal effusion (of a woman) and blood is made cold and dry. And the sign is that the menstrual blood is delayed and limited.^57^ And it is red and thin (diluted). And when it (menstrual blood) starts flowing, and if it is a trifle at a time, it is also delayed/disjointed by a few days because the blood is (affected by) phlegm that is not being expelled quickly. Her hair starts thinning. If the cold temperament takes over the entire body, the colour of the person becomes pale/white and every part touched is cold. Apart from that, whatever coldness is accompanied (by the disease) is visible.

Cure: If the perfect temperament is normal, a calefacient (*muskhanat*) is needed. If the matter is phlegm, primarily you need an emetic (*mustafrigh*). And take medicines for removing bad air, and (*ḥaqneh-jāt*) medicines for removing bad blood/urine. And then to transform (the humour), it is useful to consume the following: *masrūdīṭūs* (antidote for removing toxicity)*, sanjarnaya* (opiate-based medicine), and medicine of *al-musk* should be consumed. And take saffron, hyacinth (*sanbal*), *iklīl* (A[n] herb smelling like frankincense, rosemary), *sazāj-i hindī* (lit. Indian leaf/spikenard), and the fat of duck and chicken, egg yolk, and oil of Nardin need to be blended completely and consumed. It is put on a piece of wool which is put on the body (*za badan ālūdeh*) as a suppository. And once the woman is purified after menstruation, red arsenic, myrrh, the fruit of the cypress tree, gum, galbanum, and pills should be fumigated (*tabkhīr*) in a sack.^58^

Here, Arzanī writes that because of the woman’s cold temperament, the female seed and blood become cold. This is different from Jāmī, who indicated that there was no seed within the confines of a cold womb, and thereby the *male* semen became ineffectual. This indicated that while there was a shared, inter-genre humoural understanding of the causes of barrenness, speculatively even some measure of intertextuality, their understanding of all matters was not always congruous, nor were they meant to be so. Here, the irregularity of the woman’s menses, combined with the discolouration of the discharge, was indicative that coldness was the cause of the disease. These signs could be judged only by either checking or speaking to the patient about intimate matters.

On the other hand, Arzanī also gives a list of signs visible to any observer, such as discolouration of the skin and hair loss, combined with a full-bodied coldness. Taking the reader deeper into the humoural analysis, he clarifies that these could be caused by unrelieved ‘*balgham’* or phlegm, viscous moisture or mucus readily evident in various discharges of the body. Arzānī’s cure strikes right to the heart of the problem by suggesting a *mustafrigh*, an emetic, a substance which would cause the removal of phlegm by vomiting. Another possible solution could be to give the patient a *masrūdīṭūs* (lit. invented by Mehrdad/ Mithridates), which was an antidote against any form of toxin in the body. While he does not go into the components of these compound medicaments, they were supposed to rid the body of coldness and restore constitutional balance.

Another recipe suggested by Arzānī (as part of the same cure, not translated here) included saffron, hyacinth flowers, egg yolk, and a range of spices such as different types of spikenard, to be cooked in duck and chicken fat as well as the oil of Nardin. Indian spikenard is well-known in medications for ‘cold’ diseases: it appears in a recipe for the treatment of obstruction, wind, diarrhoea and pleurisy, and was meant to ‘arouse sexual desire … and cures the womb’.^59^ Similarly, the oil of Nardin, also a synonym for spikenard, was known for stopping bleeding in the womb and improving fertility.^60^ Most of these ingredients had heating properties and were meant to induce heat in the body. Certain ingredients had dual purposes; for instance, egg yolk was presumably meant to act as a binding agent. Much more detailed than Jāmī, he prescribes one recipe to consume orally and the other as a suppository to be inserted after menstruation, leading to the purification of the body. Arzānī’s detailed description advises the patient to combine and burn ingredients intended to combat coldness, such as arsenic, myrrh and gum. He then adds elaborate instructions to ensure that the fumes of this mixture are directed towards the ailing woman’s vagina, as this was meant to be beneficial in altering the effects of the disease.^61^

Furthermore, physicians like Arzānī were aware that coldness could persist even after the woman was successfully pregnant. To combat it, he included advice in another eighteenth-century text attributed to him, *Shifā-us ṣibyān* [Remedies for Children]. He wrote ‘if the pregnant lady is having palpitation due to cold morbid material, then it will be cured by taking lukewarm water, Rose and performing exercise. Most of the time, the pregnant lady should be given the following *Muqawwiyat-e-Qalb* (cardiac tonics) drugs so that the foetus can get vigour e.g., *Mufarrehat-e-yaqooti, Triyaq Masroodeetoos, Dawaul Misk Moatadil, Behmanain, Daroonaj*, *Zaranbad*, etc.’^62^ Clearly, medical theoreticians included solutions for a problem across pre- and post-foetal formation and provided detailed cures for all situations.

Lastly, a deeper look at the ingredients, especially the ones containing meat, leads to questions about the use of these medicines by certain classes and religious groups. The universal suitability or use of medical properties can be called into question as ‘dietetic beliefs were multifarious in character and often varied between different social groups and communities’.^63^ It would be difficult therefore to map each ingredient onto early modern consumption patterns without a deeper study. In early classical Ayurvedic medical texts the consumption of meat, either as part of diet or medicinal preparation, is without concern. But the use of meat becomes an issue for the later commentators until vegetarianism becomes a central focus of ethical arguments against meat consumption even as a curative substance and most animal products are replaced by vegetable and mineral substances.^64^ And yet, the continued prescription of animal products in the popular sixteenth-century Ayurvedic text *Bhavāprakaśa*, indicates that animal meat as well as their private organs continued to be prescribed as medicines, especially for increasing virility, and their consumption pattern crossed religious, gender, and class divides.^65^

## Fragmented knowledge: The *Ilāj-i ʿAūrat* and its prescriptions for infertility

While the *Ṭibb-i Akbarī* was a popular medical handbook, with a long pedagogical afterlife in colonial India,^66^ in the eighteenth century, another category of *ṭibbī* texts seems to emerge that combined prescriptions from across medical traditions, seemingly in random order, especially on sexual diseases of men and women. These texts are usually anonymous and bring together simpler recipes to combat issues ranging from impotency and infertility to sexually transmitted diseases. One such text is the **ʿ** *Ilāj-i **ʿ**aūrat*, which is an anonymous late-eighteenth-century text on the diseases of women. The extant work comprises two *bābs* (chapters) extracted from an unnamed mid-eighteenth-century medical work. The first chapter is about women, concentrating on prescriptions for barrenness or preventing miscarriages. The other chapter is a mix of amulets and *ṭilisms* (magical charms), ranging from concerns about procreation to finding one’s desirable mate. The following excerpt is from the first section (*faṣl*) of the first chapter, on ‘Curatives for barrenness (*‘aqīmeh*) and taking of the womb (*ḥamal giraftan*)’. A cursory look at the recipes shows that in comparison to the aforementioned prescriptions from *Laẕẕat al-nisā’*and *Ṭibb-i Akbarī*, this text spends little or no time describing the disease, except to state its name. There is no mention of the symptoms of the disease. In terms of curatives, simpler ingredients were prescribed, including the quantities and the time when they must be consumed. It could be speculated that, by the end of the eighteenth century, these were meant for individuals with some medical training and/or who could use the practical information encapsulated in the texts to create their own curatives. In the **ʿ** *Ilāj-i **ʿ**aūrat*, there are several short prescriptions for curing barrenness, but their cause for infertility is never mentioned. For instance, the author writes:

*Nau digar: Bīyārad iskandeh yak dirham shakar tar dō dirham roghan-i gāv yak dirham yak-jā kardeh hamīn qadr zanī ‘aqīmeh har roz tā deh roz bekhurd bar gīrad.^67^*

Take one dirham of *iskandeh* (garlic), two dirham of *shakar tar* (sugary water), one dirham of *roghan-i gāv* (cow’s ghee), these should be brought together (*yak-jā kardeh*). This much the barren women (*‘aqīmeh*) is supposed to consume it every day for ten days.

The contrast between this prescription and those from *Ṭibb-i Akbarī* or even *Laẕẕat al-nisā’* is marked. The prescription is simpler and uses household items like ghee, garlic, and sugary water. Unlike *Ṭibb-i Akbarī* (but similar to *Laẕẕat*), the author here states the exact quantities of each ingredient. While the author does not mention the cause of barrenness explicitly, it could be speculated from the ingredients that this prescription might also be to counter coldness in the womb. Combining garlic with ghee would counter the humoural imbalances stemming from a cold temperament (*mizāj*) in the body. As mentioned earlier, garlic, an emmenagogue, was meant to induce heat in the body, as it was considered by medieval Arabic physicians to be a hot and dry drug, used to cure diseases of the stomach.^68^ Here, presumably, ghee is simply a means of holding the ingredients together, but its effect was to increase the absorption of the cure in the stomach.

Lastly, the ‘*Ilaj-i **ʿ**aurat* provides the reader/user with the exact timeframe in which they should consume the prescription. This pattern is replicated in most of the other prescriptions in this text. Clearly, an argument for gradational readership practices is reinforced when one compares ‘*Ilaj-i **ʿ**aurat* with the more elaborate *ṭibbī* manuals or the succinct eroto-medical *Laẕẕat al-nisā’.* Even though all three types of texts are collating and imparting medical knowledge about infertility, how they do so, their narrative structures, and their details are completely different. This suggests that while some measure of medical understanding was necessary for using all three types of texts, each had a dedicated (if overlapping) audience.

In this differentiated consumption I situate the difference wrought by early modernity in medical reading practices. I argue that the major difference in eighteenth-century North India is that, in the case of feminine ailments, there is an entirely new genre of textual sources under our purview, such as the **ʿ** *ilm-i **ʿ**aūrat* concentrating on ‘the medical knowledge of women’, particularly pregnancy and childbirth. These were generally small, targeted texts with knowledge of what is today considered gynaecology and obstetrics. The **ʿ** *Ilāj-i **ʿ**aurat*, and even *Laẕẕat*, seem to be practical medical guides whose purpose was to assist their readers, medical professionals or laymen, in identifying and understanding the disease, usually by giving the most specific ‘*alamat* or sign for the disease within the heading. The other, more ephemeral purpose was to indicate the exact quantity of the ingredients and the process of creation, however brief, which would potentially allow the reader to produce a variety of these remedies in their own households. This would allow them not only to theoretically know what the problem was but actively seek a remedy for it.

## View from a *ḥakīm*’s case files: The *Maṭab-i Ḥakīm* ʿ*Alavī Khān*

While all of the aforementioned texts gave us a perspective of early modern medical knowledge production, none can offer the perspective provided in the *Maṭab-i Ḥakīm* **ʿ** *Alavī Khān*, the purported case files of an eighteenth-century Mughal physician, Ḥakīm **ʿ**Alavī Khān Shirāzī, who practised in Delhi.^69^ He included accounts of how he treated individual ailments on a case-by-case basis. While there is a high possibility that these were idealised cases, from his text one can assume that the physician would not just prescribe one remedy, but there would be a constant back-and-forth between the physician and the patient that, in the case of women, was sometimes mediated by the *qābila* (an experienced female medical practitioner)^70^. To provide a well-rounded picture of existing medical practices, I will now move beyond locating/translating fertility problems caused by cold. Instead, consider the details of a case of a woman who has the problem of ‘frequent miscarriages’:

*Ek khātūn ko isqāṭ-i ḥamal ki **ʿ**ādat thī. ḥakīm ṣāḥib ne darj ẕail **ʿ**ilāj tajwīz kiyā: dūqū 3 māshe, makōh 6 māshe, parsiyā-au shān, tukhm karfasī, zīreh siyāh har ek se 4 māshe, post falūs, khīyar- shanbar 4 tōla, khar-i khushk 6 māshe, tamām dawaūn ka jōshānda tayyār kar lein aur sharbat bezūrī 6 mashe ke humrah ist‘amāl karein- dūsre dīn dard kam ho gayā – ḥakīm ṣāḥab ne choṯ ka andīsheh ẓahir kiyā aur farmayā keh dard ke vasṭe ‘arq bādiyān 5 tōleh, gulāb 6 tōla, nosh kareīn – us ke b**ʿ**ad qābila ne isqāṭ ki dawā dī, khūn bahut nikla – kokh pāiṯ aur pehlū tak dard pahunch gaya- ḥakīm ṣāḥab ne faṣd tajwiz kiya, us ke b**ʿ**ad saqṭ ho gaya- albatta bukhār chaḍh gaya- ḥakīm ṣāḥab ne tashkhīṣ kī keh raḥim mein waram se aur isqāṭ ke b**ʿ**ad **ʿ**amuma waram ā jatā hai jisse keh zānū aur pinḍlīyon mein dard hotā hai.* ^71^

In this case, a woman had the problem of frequent miscarriages (*isqaṭ ḥaml ki ‘ādat*)^72^. The *ḥakīm* prescribed the cure which follows: three māshe seeds of wild carrot (*dūqū*), *six māshe of Solanum nigrum* (*makoh*), Maidenhair fern (*parsiya-au shan/Adiantum capillus-veneris*), celery seeds (*tukhm karfasī /Apium graveolens*), and black cumin seeds (*jireh siyah*). Take four *māshe* from each one of these, *Khīyar-shanbar* (*Cassia fistularis*), *Khar Khushk* (*caltrop/tribulus terrestris*) *six māshe*, all of these medicaments should be brought together to prepare a concentrated liquid resulting from heating or boiling these substances, a decoction. Next day, the pain had reduced – the physician examined the wound and told her to consume *five tōla ʿarq Badiyan* and six *tōla* rose for the pain. After that the qābila (*a medically experienced woman*) gave a medicine for inducing miscarriage, a lot of blood came out. The pain reached the womb, and stomach, after which the physician was consulted for the problem, who diagnosed the disease to be caused by swelling in the womb after miscarriage, which leads to pain in the knees and the calves.

The complex cure for the woman’s miscarriages provided by Ḥakīm **ʿ**Alavī Khān included celery seeds (*tukhm karfasī* or *Apium graveolens*), which were well-known in the medical community as diuretic drugs, administered to achieve the ‘expulsion of morbid matter through urination’.^73^ In this particular prescription, there is a combination of another category, *mulayyam* (laxative), which was also known to expel morbid matter, especially that concentrating around areas in the stomach, intestines, and their surroundings. Amongst the curatives mentioned in the aforementioned prescription, the laxatives singled out for curing inflammation in the viscera are *Khayarshambar* (*Cassia fistula* fruit) and *Makōh.* The humoural properties of most of these prescribed ingredients were expulsive in nature: they were meant to induce the passing of any morbid matter such as air, urine, or even in some cases semen, to clear a blockage.^74^ The case progressed when the physician noted that the day after this medicament was consumed by the woman, there was a substantial decrease in her pain levels. A close look at the prescription reveals the importance of the physician in delineating ingredients, quantities, and days of prescription. He also made observations on the wound, probably in the womb caused by repeated miscarriages, and for the pain prescribed a decoction of rosewater with ‘*Arq Badiyan*, a kind of fennel juice, a popular Unani medicament for reducing flatulence, abdominal discomfort, and restoring digestive order. This also underscores the close connection between digestion and reproduction.

It is noteworthy that the *qābila* is called in to give the abortifacient (*isqāṭe dawā*) to the patient. While little is known about the figure of the *qābila* in South Asia, they seem to be the equivalent of ‘medically experienced women’ whose testimony was legally acceptable.^75^ Her presence here could be because atters related to childbirth (or related complications) fell under her domain, as well as the fact that in intimate matters, the physician’s access to the female body was mediated by medically experienced women. Furthermore, the nature and composition of the abortifacient are not mentioned by the *ḥakīm*, yet it is possible that it was a well-known medicament, enough so that the *ḥakīm* assumed that his readership would be aware of its composition, or would have the wherewithal to consult other medical pharmacopoeias, or (speculatively) even approach an apothecarist for it. The patient bled profusely as a result of taking the abortifacient and the pain spread from the womb to the stomach and hips. The physician was seemingly wary of giving any more medicine, suggesting bloodletting (*faṣd*) would tighten the womb but lead to fever. The physician explained this fever could be stemming from the swelling of the womb, which is a possible outcome of abortions. This fever is accompanied by pain in the calf (*pinḍlīyon mein dard*).

This discussion further illustrates the sharing of knowledge in the eighteenth-century literature beyond ‘theoretical’ medical texts as pain in the calf was listed as a symptom of a problem in conceiving a foetus in the eroto-medical *Laẕẕat al-nisā’* too. There, the symptom indicating the fourth reason for barrenness is ‘*waram’*/swelling inside the uterus, a sign of which is the woman experiencing pain in the calf (*pinḍlī*) after sexual intercourse.^76^ The correspondences between all these different kinds of texts – ‘Alavī Khān’s case files, theoretical medical manuals written by and for physicians such as *Ṭibb-i Akbarī*, and the eroto-medical texts such as *Lazzat al-nisā’* – demonstrate their intertextuality. It strongly suggests that *ḥakīms* of a certain class were reading and circulating selected texts, and understood bodily humours similarly. Both medical and erotological literature focus on combatting infertility and fostering reproduction. Lastly, the reiteration of ways to remove impediments to pregnancy reinforces the conclusion that infertility caused individual and social anxieties in the early modern period.

## Conclusion

Through a critical study of early modern North Indian medical and erotological sources, this paper has explored how perceptions of and anxieties around childlessness are imperative in the making of feminine identities. By mapping the multi-linear translation, transmission and transformation of medical knowledge from Sanskrit to Braj Bhasha and Persian, I have highlighted the diverse practices to cure feminine infertility. Each section has dealt with different types of sources to underscore how generic conventions shape our understandings of medical knowledge. Each genre brought its own narrative structure and details to cure infertility in women. And yet, it is difficult to delineate if women had access to these texts or the requisite medical information to cure themselves. This ensured that cures for female infertility were mediated by male physicians who, especially in the case of elite women, did not even have unmitigated access to their bodies. While they did not have unfettered access to their subjects, early modern North Indian erotological and theoretical medical authors were aware of contemporaneous humoural models and two-seed generative model theories. Generic conventions dictated the range and details of diagnostic and curative processes on offer. On one hand, the *Laẕẕat* tradition summarised the seven-fold causes of impediments to conception, ranging from medical to magical. On the other, the formalised, theoretical *ṭibbī* tradition offers a range of diagnostic scenarios, each fleshed out in terms of the cause, **ʿ** *alamat* (symptoms), and the **ʿ** *ilāj* (cure). Within the broader medical genre, the physician’s case files or *Maṭab* show how the disease was individualised and how the patients’ experiences found their way into records of medical knowledge. It also offers precious insight into the interactions between a physician and a woman with specialised medical knowledge (*qābila)*; especially how they came together to clash or combine knowledge, skills, and access to the female patient’s body to treat her. All this (in itself) was nothing extraordinary as the desire for a child spans centuries, but it is in the eighteenth century that we see the emergence of a ‘new’ type of sub-genre within medical texts: fragmented, (mostly) anonymous, specialist texts on the cures for women. They succinctly brought simpler prescriptions to the reader, possibly allowing them to be made within the household by anyone with limited medical understanding, which fundamentally alters what we know of early modern feminine medical spaces and practices.

This article provides a preliminary overview into the different tiers of medical theoreticians and practitioners who provided diagnostic and curative services as well as actively produced medical knowledge through writing (and commenting). While I have tried to show the existence of female medical practitioners, the eighteenth-century anonymous prescription texts hint at the presence of a more ephemeral group of readers – the women within the *harem* with medical knowledge. Speculatively, some of the ‘simpler’ cures, with accurate measurement of ingredients, recipes, and timelines for consumption, were pitched at these groups. This understudied nexus of medical knowledge transfer reveals as much about the early modern medical marketplace as it does about issues of sexual medicine considered relevant for those who desired to bear a child.

Much like their Western European counterparts, early modern North Indian erotological and medical texts also emphasised generating heat and pleasure to foster fecundity and fertility, usually by prescribing aphrodisiacs. In the case of women, generally, this advice was couched in prescriptions to fight barrenness. This article followed how to cure barrenness caused by a ‘cold womb’ through various textual genres. Each genre, with varied degrees of detail, provided cures to restore the humoural balance by prescribing cures to generate heat in the body. Lastly, while the medical texts fulfilled a diagnostic and curative purpose, my article demonstrates that erotological texts like *Lazzat- al nisā’* not only contain information about desired codes of sexual conduct but also noted ways to fulfil the generative potential of the Timurid-Mughal elite. This elite household was a central feature of early modern society, as well as a site for showcasing male and female fecundity. As bearers of children, the evidence of fertility marked a woman’s body. A childless woman, while not without power, remained a potential problem. By the eighteenth century, as marriage without children could not be envisaged, the inability to produce offspring remained central to the socio-sexual anxieties of early modern South Asian women.

## Footnotes

1 Cf. B.S Musallam, *Sex and Society in Islam: Birth control before the nineteenth century (*Cambridge: Cambridge University Press, 1983), 142, f.n. 76.

2 This vignette from Timurid-Mughal Emperor Jahangir’s (r. 1605–27) memoir underlines how childlessness and the pursuit of progeny cut across class boundaries. Wheeler Thackston (trans., ed. and anno.), *The Jahangirnama: Memoirs of Jahangir, Emperor of India* (Oxford: Oxford University Press & Freer Gallery of Art, Smithsonian Institution, D.C., 1999), 21.

3 Most of my sources were composed in the seventeenth- and eighteenth-century North Indian territories of the Mughal empire stretching from Kabul to Benares. They are primarily composed in Persian, which had courtly patronage and was the language of elite knowledge production. Towards the end of the eighteenth century, we see a proliferation of a lower register of Persian in the medical sources as well as the nascent rise of Urdu. For a detailed account see Christopher King , One Language, Two Scripts: The Hindi Movement in Nineteenth Century North India (New Delhi: Oxford University Press, 1994). Some sources are in Braj bhashā, one of the most important vernaculars spoken in the multilingual early modern North Indian milieu. See Allison Busch , Poetry of Kings: The Classical Hindi Literature of Mughal India (London: Oxford University Press, 2011).

4

Ruby Lal , Domesticity and Power in the Early Mughal World (New York: Cambridge University Press, 2005), 188 .

5 For a broad overview, see J.F Richards , The Mughal Empire (Cambridge: Cambridge University Press, 1993).

6 For a comprehensive survey, see Michael H. Fisher , A Short History of the Mughal Empire (London/New York: I.B Tauris, 2016), and Tapan Raychaudhuri and Irfan Habib (eds) *The Cambridge Economic History of India (1200-1750)*, Vol.1 (Cambridge: Cambridge University Press, 1982).

7

Lisa Balbanlilar , Imperial Identity in the Mughal Empire: Memory and Dynastic Politics in the Mughal Empire (London: I.B Tauris, 2012), 102 .

8 For a comprehensive survey on the Mughal harem, see Lisa Balballinar , ‘The Begims of the Mystic Feast: Turco-Mongol Tradition in the Mughal Harem’, The Journal of Asian Studies, 69:1 (2010), 123–47, 137; Ruby Lal , ‘Historicizing the Harem: The Challenge of a Princess’s Memoir’, Feminist Studies, 30:3 (2004), 590–616 ; and Taymiya R. Zaman, ‘Visions of Juliana: A Portuguese Woman at the Court of the Mughals’, *Journal of World History*, 23:4 (2012), 761–91.

9

Munis Faruqui , Princes of the Mughal Empire 1504-1719 (New York: Cambridge University Press, 2015), 24–26 .

10 Shah Jahan was entrusted to the care of Emperor’s Akbar’s chief wife, Ruqayya-Sultan Begum, who was childless. Thakston, *Jahangirnama*, 437. Furthermore, the fact that the child was adopted did not have any impact on the adoptive mother’s already substantial power and influence, nor on the potential political legitimacy of the fostered princes. See Lal, *Domesticity and Power in the Early Mughal World*, 188–96.

11 Furthermore, she would be a free woman at the time of the master’s death. In the Shi‘as, she would not be sold during the life of her child, and her right to emancipation depended on her child’s share in the estate. Cyril Elgood , Safavid Medical Practice, or The Practice of Medicine, Surgery and Gynaecology in Persia between 1500 A.D. and 1750 A.D. (London: Luzac &Co., 1970), 199.

12 Lal, *Domesticity and Power in the Early Mughal World*, 128–37; and Afshan Bokhari, ‘Gendered “Landscapes”: Jahan Ara Begum’s (1614–81) ‘Patronage, Piety, and Self-Representation in the 17^th^ century Mughal India’ (unpublished PhD thesis: University of Vienna, 2009), 50-71.

13 Lal, *Domesticity and Power in the Early Mughal World*, 128–30.

14 The twenty-first chapter on the diseases of women in the eighteenth-century theoretical medical handbook, *Ṭibb-i Akbarī*, is titled ‘special diseases of women’ (*ān dar bīmārī-hā keh zenān makhṣūṣ ast*), Muḥammad Akbar Arzānī, *Ṭibb-i Akbarī*, British Library, IO Islamic 2017 [1718], f. 278a, and for the Urdu version Muḥammad Akbar Arzānī, *Tibb-i Akbarī Urdu* (Lucknow: Munshi Naval Kishor, 1880), f. 439.

15 These have long been considered to be a triumvirate of neighbouring Muslim Asiatic empires. There have been a spate of comparative histories of these early modern Muslim polities, a few of them are G. S Marshall Hodgson , *The Venture of Islam: Conscience and History in a World Civilization*, Vol.1 (Chicago: University of Chicago Press, 1974); N. R. Farroqi , Mughal-Ottoman Relations: A Study of Political and Diplomatic Relations between Mughal India and the Ottoman Empire 1556-1748 (Delhi: Idarah-I Adabiyat-I Dihli, 1989); Stephen F. Dale , The Muslim Empires of the Ottomans, Safavids and Mughals (Cambridge: Cambridge University Press, 2010); and Douglas Streusand, *Islamic Gunpowder Empires: Ottomans, Safavids, and Mughals* (London: Avalon Publishing, 2010).

16 Since Marshall Hodgson coined the term ‘Persianate zone’ for his three-volume work *The Venture of Islam: Conscience and History in a World Civilization*, the concept has evolved into what Nile Green calls the ‘Persianate hypothesis’. This was supposed to be the long-term ‘dominance *qua* influence of Persian over the other languages and cultures of the eastern Islamic world’. Nile Green (ed.), *The Persianate World: The Frontiers of a Eurasian Lingua Franca* (Oakland, CA: University of California Press, 2019), xiii. For the South Asian specificity, see Muzaffar Alam, ‘The Pursuit of Persian’, *Modern Asian Studies*, 32:2 (1998), 317–49; and John Seyller , Workshop and Patron in Mughal India: The Freer Ramayana and Other Illustrated Manuscripts of ’Abd Al-Rahim (Zurich: Artibus Asiae Supplementum 42, Rietberg Museum, 2007).

17

Hasan Askari , ‘Medicines and Hospitals in Muslim India’, Proceedings of the Indian History Congress, 20 (1957), 170–83; Fabrizio Speziale, ‘Persian Treatises on Āyurveda: The Shaping of a Genre’, *Journal of South Asian Intellectual History*, 3:1 (2021), 89–122; and Seema Alavi , Islam and Healing: Loss and Recovery of an Indo-Muslim Medical Tradition, 1600-1900 (Ranikhet: Permanent Black, 2007), 28–42 .

18 Shalini Shah, ‘Representation of Female Sexuality in the Ayurvedic Discourse of the Early Medieval Period’, *Studies in History*, 22:1 (n.s.) (2006), 45–58; and Martha Ann Selby, ‘Narratives of Conception, Gestation, and Labour in Sanskrit Ayurvedic Texts’, *Asian Medicine*, 1:2 (2005), 254–75. Also see K. Bharathi and V.V.L. Prasuna , ‘Ancient Indian Knowledge of Maternal and Child Health Care: A Medico-Historical Introspection of Ayurveda’, Journal Indian Medical Heritage, 39 (2009), 221–40.

19 For more on *ṭibb* in early modern South Asia, see Shireen Hamza , ‘Vernacular Languages and Invisible Labor in Tịbb’, Osiris, 37 (2022), 115–38; Deborah Schlein, ‘In the Ḥakīm’s Own Hand: A Study of Colophons and Ownership Notes of the al-Asbāb wa-l-ʿalāmāt Commentary Tradition in India’, *Journal of Islamic Manuscripts*, 9 (2018), 264–71; and Alavi, *Islam and Healing*, 28–42.

20 There is some inconsistency in the number of days between the Persian edition (*chahal*, forty) and the English translation (fifty). I have chosen to follow the former. See Syed Moinul Haq (ed.), *Dhakhirat al-Khawanin of Shaykh Farid Bhakkari*, Vol. 2 (Karachi: Pakistan Historical Society, 1970), 245. For the English translation, see Z.A Desai , Nobility Under the Great Mughals: Based on Dhakhiratul Khawanin of Shaikh Farid Bhakkari (New Delhi: Sundeep Prakashan, 2003), 83 .

21 A recent study reaffirms that a legitimate line of paternity was a concern across classes. For instance, in early modern Mewar, children of uncertain caste resulting from inter-caste live-in relationships (even amongst non-elite groups) would have led to disputes over property inheritance. See Divya Cherian , ‘Stolen Skin and Children Thrown: Governing Sex and Abortion in Early Modern South Asia’, Modern Asian Studies, 55 (5), 1461–1509 , 1485.

22 Even though there is no mention of a one- or two-seed model of procreation here, the dominance of the male traits that the dead man passed on to his progeny through food was implicit in the story. Once consumed, food is cooked by the digestive fires and through various processes transformed into blood, leading eventually to the highest essence generated by the (male) body: semen. The link between food’s effects on semen through the digestive process has been discussed both in Ayurvedic and Yunani sources. See Dominik Wujastyk, ‘The Science of Medicine’, in Gavin Flood (ed.), *The Blackwell Companion to Hinduism* (London: Wiley Blackwell, 2003), 398–99.

23

V.B. Dash , and V.L Kashyap (trans. and ed.), Materia Medica of Ayurveda based on Ayurveda Saukhyam of Todarnanda (New Delhi: Concept Publishing Company, 1980), 286–87.

24

Martha Ann Selby , ‘On Anatomical Enumeration and Difference in Early Sanskrit Medical Literature’, Asian Medicine 14 (2019), 274 .

25 He considered *rohu* to be the ‘best fish in Hindustan’ and even gifted a horse to the commander of the foot soldiers in his retinue, Rai Man, for catching a *rohu* fish for him while they were on the road. Thackston, *The Jahangirnama*, 239, 242, 416.

26 Meat and alcoholic drinks featured heavily amongst Ayurvedic therapies, even though most theorists thought that it was against ethical norms. Clearly, the desire to restore health (or potency) ensured that people consumed medicaments which ran directly counter to a dietary rule against meat, blood, and claws of animals formulated repeatedly in *śāstras* (codes), and epics. See Francis Zimmerman , The Jungle and the Aroma of Meats: An Ecological Theme in Hindu Medicine (Delhi: Motilal Banarsidass Publishers, 1999), 159–8310.1016/0277-9536(88)90121-93175704, 185.

27 Jean Baptiste Tavernier, *Travels in India*, V. Ball (trans.) and William Crooke (ed.) [2 vols. bound in one] (New Delhi: Low Price Publications, 2000) (first published, London, 1925), Vol. I, 61–2.

28 Recent research shows that medical texts and authors were patronized by the Mughal state and thus contributed to building political sovereignty and impacted contemporary society and culture in longer-term ways. They laid down norms of civility that continued to be negotiated even after the collapse of the Mughal empire. Alavi, *Islam and Healing*, 4.

29 For more on *tridoṣa- vidyā* or the three semi-fluid humoral substances which are present in the body, see Dominik Wujastyk (ed. and trans.), The Roots of Ayurveda: Selections from Sanskrit Medical writings (New Delhi: Penguin Classics, 1998), 3–8 . For more on the Greco-Arab system, see Peter E. Pormann and Emilie-Savage Smith (eds), Medieval Islamic Medicine (Edinburgh: Edinburgh University Press, 2007), 43–5; and Manfred Ullmann, *Islamic Medicine* (Edinburgh: Edinburgh University Press, 1978), 51–8.

30 Speziale demonstrates that certain Yunani scholars proposed to revise and adjust the Greco-Arabic view of humoral pathology for Muslim physicians practising in the Indian setting. For instance, in the fourteenth century, Shihāb al-Dīn Nāgawrī composed a medical handbook titled Shifāʾ al-maraż [Healing of Disease] to integrate the Ayurvedic concept of wind (*vāta* in Sanskrit, *bād* in Persian) into the fourfold division of humoral pathology of Muslim physicians. See Fabrizio Speziale , ‘A 14th Century Revision of the Avicennian and Ayurvedic Humoral Pathology: The Hybrid Model by Šihāb al-Din̄ Nāgawri’, Oriens, 42 (3–4) (2014), 514–32.

31 Two rare recent interventions which utilized *Laẕẕat al-nisā’* are Suzanne Kurz , ‘Never just for fun? Sexual Intercourse in Perso-phone Medicine, Erotology, and Ethics’, in Suzanne Kurz , Claudia Preckel , Stefan Reichmuth (eds), Muslim Bodies: Body, Sexuality, and Medicine in Muslim Societies (Berlin: Lit Verlag, 2016), 97–130 ; and Sajjad Rizvi , ‘This So Sullied Flesh? Islamic Approaches to Human Pleasures’, in Lisa Shapiro (ed.) Pleasure: A History (Oxford University Press, 2018), 66–93 .

32 There is a large number of erotological works that use the word *koka.* These treatises are named *Koksār*, *Kokśāstra*, *Kokmañjarī*, *Kokvidhi*, *Kokvilās*, *Madankok*, or *Raskok.* For more, see Nadia Cattoni , ‘The *Koksār* by Ānand Kavi: A Popular Erotic Book’, in Imre Bangha , and Danuta Stasik (eds), Early Modern Literary Cultures in North India—Current Research (Oxford: Oxford University Press, 2024), 259–78.

33 Muḥammad Jāmī, *Laẕẕat al-nisā’*, National Museum, Delhi, Acc. 85.351 [1646]. This version is illustrated with high-quality Deccani paintings but is incomplete.

34 Another possible author of the text is the famous Sufi writer, Żiyā’ al-Din Nakhshabī (d.1350–51), although no known manuscript survives from before the seventeenth century. Żiyā’ al-Din Nakhshabī was a fourteenth-century Chisti mystic and author whose family emigrated to India from Nakshab (Iran). He might have been a practitioner physician. His reputation is mainly due to the books he wrote in Persian or translated from Sanskrit, and the intervening Sufi concepts throughout his mystical works, religious, and literary works. For *Laẕẕat al-nisā’*s tradition attributed to Żiyā’ al-Din Nakhshabī, see Suzanne Kurz, ‘“Laḏḏat al-nisā”’, Perso-Indica: An Analytical Survey of Persian Works on Indian Learned Traditions, F. Speziale - C. W. Ernst (eds) (2018), available online at http://www.perso-indica.net/work/kokasastra_%28laddat_al-nisa%29.

35 For more, see Sonia Wigh, ‘A Body of Words: A Social History of Sex and the Body in Early Modern South Asia’ (unpublished PhD thesis: University of Exeter, 2021).

36 There are various traditions and recensions in the *Kokaśastra* corpus. This citation is not indicative of longer, more complex prescriptions found in other recensions. Cf. Alex Comfort , Koka Shastra, being the Ratirahasya of Kokkota, and other medieval Indian writings on love (London: George Allen & Unwin, 1964), 168 .

37 While the ingredient is *Nāgakesara*, the ‘*nava’* could be indicative of new or young roots of the plant. Uday Chand Dutt , The Materia Medica of Hindus (Calcutta: Adi-Ayurveda Machine Press, 1922), 120, 311.

38 Anand Ram Mukhliṣ in his *Mir**ʿ**āt al-iṣtilāḥ*, mentioned that he had never seen it as its flower bloomed once in seven years, it was well-known for its fragrance and had to be brought in from Bengal. While the price of the roots is unknown the price of its perfume in the Deccan during the reign of Emperor Aurangzeb was one hundred rupees for a *tola.* See Anand Ram Mukhlis, *Mirat-ul Istilah: Encyclopaedic Dictionary of Medieval India* Tasneem Ahmad (trans.) (Delhi: Sundeep Prakashan, 1993), 201.

39 Dash and Kashyap (trans. and ed.), *Materia Medica of Ayurveda*, 173.

40 Ibid, 141–2.

41 Muḥammad Jāmi, *Laẕẕat al-nisā’*, National Museum (New Delhi), Acc. 85.351 [1646], f. 20b.

42 Muḥammad Jāmi, *Laẕẕat al-nisā’*, British Library, Add. Mss.17489 [1797] ff. 29b–31a.

43 The Islamic tradition generally holds that Solomon was the third King of Israel and a just and wise ruler of the nation. Islam views Solomon as one of the elects of Allah, who was not only wise beyond measure but could speak to animals and control spirits. It is also noteworthy that according to the Quran, Solomon would always be associated with *Hikmah* (Knowledge/Wisdom), so much so that he was referred to as ‘*Sulaiman al-Hakim*’ (Solomon the Wise). See *Encyclopedia of Islam*, Vol. 2., 2^nd^ edition, P. Bearman, Th. Bianquis, C.E. Bosworth, E. van Donzel and W.P. Heinrichs (eds) (Leiden: Brill, 1991), 297.

44
*Siyāh daneh* could also be used for coriander seeds. Even in that scenario, it was meant to induce heat as all parts of the plant were considered a hot and dry drug. For more, see Efraim Lev, and Zohar Amar, *Practical Materia Medica of the Medieval Eastern Mediterranean According to the Cairo Genizah* (Leiden: Brill, 2008), 156–7. Furthermore, an alternative to this line is ‘*Bij/Buj wa panj pur az siyah dāneh khūb*’, where *bij* could be rice, while *buj* is goat. Muḥammad Jāmī, *Laẕẕat al-nisā’*, National Museum (New Delhi), Acc. 85.351 [1646], f. 22a.

45
*Ser* is a unit of measurement that amounts to a weight of 15 mis̤qāl which weighed about 4.6 grams.

46 The translation is mine. Muḥammad Jāmī, *Laẕẕat al-nisā’*, British Library, Add. Ms.17489 [1797], f. 31a. The translation is mine.

47 Much like their Western European counterparts, both Yunani and Ayurvedic theorists agreed that in most cases ‘heat not only determined the strength of a person’s sexed identity but also determined the strength of their sexual desire. Cold men and women lacked desire and were often unable to reproduce successfully, while warm bodies had a stronger sex drive and were more procreative.’ Jennifer Evans , Aphrodisiacs, Fertility and Medicine in Early Modern England (Woodbridge: Boydell & Brewer Press, 2014), 53 . For similar Islamic understanding, see Shireen Hamza , ‘Medicine Beyond Doctors: Aphrodisiac Recipes in Tenth-Century Medicine and Cuisine’ Medieval Feminist Forum, 53:2 (2017), 91–113 .

48 For a discussion on the historical evolution of the concept of ‘seed’ and female secretions, see Ahmed Ragab , ‘One, Two, or Many Sexes: Sex Differentiation in Medieval Islamicate Medical Thought’, Journal of the History of Sexuality, 24:3 (2015), 428–54, 434; and Pernilla Myrne , Female Sexuality in the Early Medieval Islamic World: Gender and Sex in Arabic Literature (London: I.B Tauris, 2020), 32–4.

49 Black cumin was meant to be superior to its *kermānī* (caraway) and *sabz* (green) variants. Hūšang Aʿlam, ‘Cumin’, *Encyclopaedia Iranica*, Vol. VI, Fasc. 5, 452–4, available online at https://iranicaonline.org/articles/cumin-pers, accessed online on 21 July 2017.

50 Lev and Amar, *Practical Materia Medica of the Medieval Eastern*, 166–7.

51 Alavi, *Islam and Healing*, 28.

52 The *Tibb-i-Akbarī* is the Persian commentary on the Arabic text *Sharḥ al-Asbāb wa al-‘Alāmat* of Nafis bin Aiwaz Kirmani (d. 1449), which itself was a commentary on Najīb al-Dīn al-Samarqandī’s (d.1222) *Al-Asbāb wa al-‘Alāmat* (Aetiology and Signs). In the early eighteenth century, Arzānī constructed and added his commentary to Kirmani’s text. For more, see Elgood, *A Medical History of Persia and the Eastern Caliphate* 303-04. Also, Deborah Schlein , ‘In the Ḥakīm’s Own Hand: A Study of Colophons and Ownership Notes of the *al-Asbāb wa-l-ʿalāmāt* Commentary Tradition in India’, Journal of Islamic Manuscripts, 9 (2018), 264–71.

53 Speziale, ‘Persian Treatises on Āyurveda’, 89–122. The link between Ayurvedic and Greco-Arabic medical traditions and how they were then interpreted by European traditions has been succinctly discussed in Biswamoy Pati and Mark Harrison (eds), Health, Medicine and Empire: Perspectives in Colonial India (New Delhi: Orient Longman, 2001). The most quoted information is from *Atharvaveda*, one of the four major Vedic scriptures likely compiled as a *Veda* contemporaneously with *Samaveda* and *Yajurveda* in about 1200–1000 BC. It contains spells for healing various illnesses, spells for the removal of demons, love spells, and material relevant to *gṛhya* (domestic) rituals, such as marriage, initiation, and death. It is difficult to attribute the growth and transmission of the genre to a particular author, but several compendia of medicines were collated by learned men and these were widely circulated and consumed. For more information, see Kenneth Zysk , Religious Medicine: The History and Evolution of Indian Medicine (New Brunswick: Transaction Publishers, 1993).

54 Incidentally, this is similar to its Ayurvedic counterparts, Selby, ‘Narratives of Conception’, 257.

55 The focus on male impotency and childlessness is discussed in detail in Wigh, ‘The Body of Words’, 293–347.

56 As Mughal eunuchs were identified as male, it is noteworthy to highlight the difference in the case of eunuchs who were physically distinguished from men by three general features: a lack of facial hair, an unbroken voice, and the inability to have children. Emma Kalb, ‘Slaves at the Center of Power: Eunuchs in the Service of the Mughal Elite, 1556-1707’ (unpublished PhD thesis: University of Chicago, 2020), 23, 26.

57
*Haiz* is both the process of menstruation and also the menstrual blood, which is one of the wastes of the body, like urine, stool and sweat. Guy Attewell, ‘Authority, Knowledge and Practice in Unani Ṭibb in India (c.1890-1930)’ (unpublished PhD thesis: SOAS, University of London, 2004), 218.

58
*Naw‘-i awwal: ān keh sawī mizāj sard dar raḥim wa uftad, wa manī wa khūn rā sard sāzad wa khushk. Wa **ʿ**alamat aū ān ast keh khūn-i ḥaiz̤ dair āyad wa kam aȳad, wa surkh wa raqīq buwad. Wa chūn āyad agarcheh andak bāshad. Amā beh zamān ṭuyīl manqaṭ**ʿ**a gardad zīrā keh khūn balghamī zaud daf‘a nashawad. Wa īn kus rā mūi dar **ʿ**āneh kam bāshad wa ānjā keh mizāj sard dar tamām **ʿ**ām buwad sapīd lūn waī sardī malmus wa juz ān keh az lawāzim barūdat ast paīda buwad.*
***ʿ**Ilāj: agar sawī mizaj sādeh buwad tabdīl ān kunand beh muskhanāt wa agar mādeh balgham būd nakhustīn mustafrigh sāzand ān rā beh aȳarjāt wa ḥaqneh-jat b‘adeh beh tabdīl ko shanad wa ān jeh badīn kār āyad mas̤rūdīṭūs wa sanjarnayā wa dava al-musk wa mānand-i ān khūrdan ast. Wa z**ʿ**āfaran and sanbal wa aklaīl wa sāzaj-i hindī wa farmānā wa paīyeh-i baṭ wa makaȳan wa zardī bīz̤eh wa roghan nārdīn jumleh beh ham āmīkhteh wa ṣauf za badān ālūdeh farzjeh sākhtan wa b**ʿ**ād az alz pāk shud tan ḥaīz̤ zarnīkh surkh wa mar wa jūz alsarū wa miy**ʿ**āh wa qateh wa ḥabb al**ʿ**ghār tabkhīr kardan.* Muḥammad Akbar Arzānī, *Ṭibb-i Akbarī*, British Library, IO Islamic 2017 [1718], ff. 286b–87a. The translation is mine.

59 Lev and Amar, *Practical Materia Medica of the Medieval Eastern Mediterranean*, 291.

60 Lev and Amar, *Practical Materia Medica of the Medieval Eastern Mediterranean*, 292.

61 Details of these ingredients and their humoural properties can be seen in Tazimuddin Siddiqi, ‘Ḥakīm Muḥammad Akbar Arzānī’, *Studies in History of Medicine and Science* (1981), 174–221.

62 Shariq Ali Khan (trans. and ed.), *Shifa-us Sibyan* (New Delhi: Asila Offset Printers for National Mission for Manuscripts, 2020), 17. I have retained the transliteration suggested by the translator. Also, Arzānī was a prolific writer and has left a substantial body of work, a synoptic view of which can be seen in Siddiqui, ‘Ḥakīm Muḥammad Akbar Arzānī’, 167 but it is unclear if *Shafa-us ṣibyān* could be rightfully attributed to him. Elgood attributes a text called ‘Ilāj al- *ṣibyān* to Arzānī, but he only cited an edition appended to the Urdu Mizan al Tibb published in Cawnpore (Kanpur) in 1866-87. C.A Storey, *Persian Literature: A Bio-Bibliographical Survey*, Vol.2, Part 2, E. Medicine (London: Luzac & Co, 1971), 268.

63 Divya Narayanan, ‘Cultures of food and Gastronomy in Mughal and Post-Mughal India’ (unpublished PhD. thesis: University of Heidelberg, 2015), 170.

64

Dagmar Wujastyk , Well-Mannered Medicine: Medical Ethics and Etiquette in Classical Ayurveda (Oxford: Oxford University Press, 2012), 133–4, 216 f.n. 435.

65

Kenneth Zysk , ‘Animal Usage in the Sanskrit traditions of Lovemaking, Lawful Conjugal Love, and Medicine’, in Nalini Balbir and Georges-Jean Pinault (eds), Penser, dire et représenter l’animal dans le monde indien (Paris: Honoré Champion, 2009), 288–303 , 297–9.

66 Alavi, *Islam and Healing*, 38–40.

67 Anonymous, ***ʿ**Ilaj-i **ʿ**Awrat*, undated (probably 18^th^ cen.), Wellcome Collection, WMS.Per.330 (B), f. 1b. The translation is mine.

68 An emmenagogue is a substance that stimulates or increases menstrual flow. On garlic, see Lev and Amar, *Practical Materia Medica of the Medieval Eastern Mediterranean*, 412–14.

69 While the original text is in Persian, I am accessing it here through a comprehensive Urdu edition. See Ḥakīm Abdul Bari Falahi (trans. and ed.), *Maṭab-i Ḥakīm ‘Alavī Khan* (New Delhi: Jamia Hamdard, 2009), xii.

70 On female medical practitioners, Avner Giladi , Muslim Midwives: The Craft of Birthing in the Premodern Middle East (New York: Cambridge University Press, 2015).

71 Bari, *Maṭab-i Ḥakīm ‘Alavī Khan*, 479.

72 The term *isqāṭ* [inf. n. iv of ’to fall, drop’] was used to denote ‘causing to fall; dropping or casting her young (an animal); miscarrying, miscarriage; procuring an abortion’. Hence, there is much ambiguity in the connotation of the term. ‘*Isqāt̤i*-*ḥamal’*, could be (and was) used interchangeably for miscarriage, and procuring an abortion. Platts, J.T Platts , A *dictionary of Urdu, classical Hindi, and English* (London: W. H. Allen & Co., 1884), 52 .

73 Siddiqui, ‘Ḥakīm Muḥammad Akbar Arzānī’, 209.

74 The purgative mentioned here, *Khayarshambar/khiyyār shanbar/ḥiyyār shanbar* (*Cassia fistula* fruit) is a powerful laxative and should not be prescribed if the patient is old, has weak intestines, or is pregnant because its expulsive faculty could provoke miscarriage. Lev and Amar, *Practical Materia Medica of the Medieval Eastern Mediterranean*, 130–2.

75 Giladi clarifies the difference between the Arabic term, *qābila*, a midwife whose wisdom is ‘born of experience’ and the Persian *dāya*, used for nurses or wet nurses. Giladi, *Muslim Midwives*, 57–8.

76 Muḥammad Jāmī, *Laẕẕat al-nisā’*, British Library, Add.17489, f. 30b. The translation is mine.

